# Efficacy of an integrated case-based learning and team-based learning model within the subjective, objective, assessment, and plan framework in physical therapy education

**DOI:** 10.3389/fmed.2026.1767677

**Published:** 2026-02-19

**Authors:** Ruike Zhang, Minglei Huang, Jinyang Xie, Yiqi Yang, Jiaqiao Yan, Zhiying Wu, Jiaxin Yuan, Adila Aisaiti, Shiyan Jiang, Zhenfeng Guo, Kai Shi, Hongxin Chen, Shuangquan Ji, Qinglu Luo, Tao Huang

**Affiliations:** 1 Department of Rehabilitation Medicine, Key Laboratory of Biological Targeting Diagnosis, Therapy and Rehabilitation of Guangdong Higher Education Institutes, The Fifth Affiliated Hospital of Guangzhou Medical University, Guangzhou, China; 2 The Fifth Clinical College of Guangzhou Medical University, Guangzhou, China

**Keywords:** case-based learning, clinical reasoning, physical therapy education, SOAP framework, team-based learning

## Abstract

**Background:**

Conventional “lecture+demonstration” pedagogy in physical therapy (PT) education frequently constrains student engagement and the evolution of clinical reasoning. The Physical Therapy for Orthopedic Diseases (PTOD) course necessitates strong clinical reasoning and collaboration, which the mixed approach of case-based learning (CBL) and team-based learning (TBL), along with the SOAP framework, aims to improve.

**Methods:**

This study employed a quasi-experimental design to analyze data collected from Guangzhou Medical University (GZMU) between March 2023 and June 2024. A total of 46 third-year physiotherapy students (2020 cohort: *n* = 25, CBL + TBL; 2021 cohort: *n* = 21, traditional teaching) completed the study. Baseline comparability between the two groups was ensured by comparing routinely collected academic performance data and questionnaire responses obtained prior to the start of the course. The 2020 cohort had participated in role-shifting SOAP activities using actual cases, whereas the 2021 cohort had received lecture-based teaching accompanied by SOAP framework. Outcomes included questionnaire scores (Self-assessment, Self-directed learning, Group-cooperation, Teacher-assessment, Learning-effectiveness, Learning-satisfaction) and academic performance (class performance, practical performance, theoretical scores) were analyzed.

**Results:**

The 2020 cohort markedly outperformed the 2021 cohort in total scores of questionnaires (*p* < 0.001) and in the sub-dimensions of Self-assessment (*p* < 0.01), Self-directed learning (*p* < 0.05), Group cooperation (*p* < 0.001), and Learning satisfaction (*p* < 0.05). In the 2020 cohort, pre-post enhancements were statistically significant in total scores (*p* < 0.001) and the corresponding sub-dimensions (*p* < 0.001). The 2021 cohort exhibited a notable reduction in group cooperation (*p* < 0.05). Overall grades indicated no significant difference (*p* > 0.05); however, the 2020 cohort demonstrated superior class performance (*p* < 0.01) and practical performance (*p* < 0.05).

**Conclusion:**

The CBL + TBL model, combined with the SOAP framework, markedly improves students’ clinical reasoning, practical abilities, and learning engagement in the PTOD course, providing a more effective alternative to traditional teaching methods for rehabilitation education reform.

## 1 Background

In the context of global population aging and the increasing incidence of chronic diseases, rehabilitation is widely acknowledged as an essential element of 21st-century healthcare strategy (1). Physical therapy (PT), a fundamental component of rehabilitation medicine, is pragmatically focused and clinically driven, designed to provide students with strong clinical skills, critical thinking abilities, and thorough clinical reasoning (2). The course of “Physical Therapy for Orthopedic Diseases” (PTOD), fundamental to rehabilitation PT programs, equips students to utilize specialized knowledge and therapeutic techniques to mitigate pain, correct abnormal physical structures, and restore function resulting from acute or chronic musculoskeletal injuries or diseases, thereby improving patient’s quality of life and facilitating reintegration into family and society (3). The current course predominantly utilizes a conventional “lecture + demonstration” pedagogical approach, prioritizing instructor-led knowledge dissemination and procedural illustrations. This strategy, long utilized in medical and clinical education (4), is proficient in structured knowledge transfer but increasingly demonstrates shortcomings in promoting profound student engagement and skill transfer. This teacher-centered approach leads to minimal student participation and restricted classroom interaction (5), impeding proactive learning. Conversely, students’ comprehension of complicated clinical issues frequently remains superficial, characterized by insufficient critical thinking and clinical reasoning abilities, which hinders their capacity to manage complex decision-making and practical responsibilities during subsequent clinical internships or professional practice (6). Moreover, the “one-way output” classroom framework restricts students to passive recipients, inadequately addressing their varied learning requirements in autonomous learning, collaboration, and the integration of theory and practice (7).

To address these challenges, recent domestic and international initiatives in PT and musculoskeletal rehabilitation education have investigated several pedagogical reform strategies, including flipped classrooms (8), e-learning (9), case-based learning (CBL), and team-based learning (TBL) (10). Research suggests that these methodologies alleviate the constraints of conventional education: flipped classrooms significantly boost student motivation (11), TBL aligns well with both theoretical and practical fields (10), CBL integrated with traditional instruction produces favorable results in practical skill evaluations (12), and e-learning paired with lectures is greatly preferred by students (13). Nonetheless, e-learning struggles to meet the high practical demands of medical courses, hindering critical hands-on training (e.g., manual techniques, bedside assessments) and resulting in underdeveloped core clinical skills (14). Flipped classrooms frequently experience insufficient involvement in pre-class activities, resulting in negligible effects on academic achievement (8). In standalone CBL, certain students encounter tensions between discussion periods and clinical practice, resulting in diminished engagement, inadequate teamwork, and insufficient knowledge consolidation (15, 16). Isolated TBL necessitates considerable preparation and reflection time, increasing cognitive load and inducing tiredness (10). Due to the substantial practical, clinical reasoning, and critical thinking requirements of PT courses, blended learning methodologies have become increasingly popular. Research indicates that blended techniques, such as the integration of computer-assisted instruction (CAI) and TBL, utilize their distinct advantages to optimize learning outcomes and performance (17). Furthermore, blended learning possesses considerable potential to enhance PT students’ attitudes and cultivate practical competencies (18).

The PTOD course is based primarily on clinical reasoning and collaboration, and the dual CBL-TBL paradigm is strongly aligned with these academic requirements. CBL connects the enduring barrier between theoretical foundations and clinical application using authentic or simulated orthopedic situations (19, 20). TBL fosters evidence-based reasoning and improves learning efficiency through organized tasks and interactive environments (5, 21). CBL and TBL collaboratively establish a synergistic cycle of “case-driven knowledge integration and teamwork enhancement,” alleviating tiredness from extended TBL talks and addressing the deficiencies of conventional teaching in promoting critical thinking (19). Therefore, to better leverage this synergistic effect, the systematic integration and optimized application of CBL and TBL in PTOD teaching have become key to improving instructional outcomes and addressing the limitations of traditional teaching methods.

Based on the aforementioned background, this quasi-experimental study aimed to evaluate the effectiveness of a blended teaching model integrating CBL and TBL compared with traditional lecture-based instruction in the PTOD course. Specifically, we examined whether this integrated approach could enhance PT students’ clinical reasoning skills, critical thinking abilities, and comprehensive practical performance, while also promoting teamwork competence and learning engagement.

## 2 Materials and methods

### 2.1 Study design and participant

**Figure 1:**
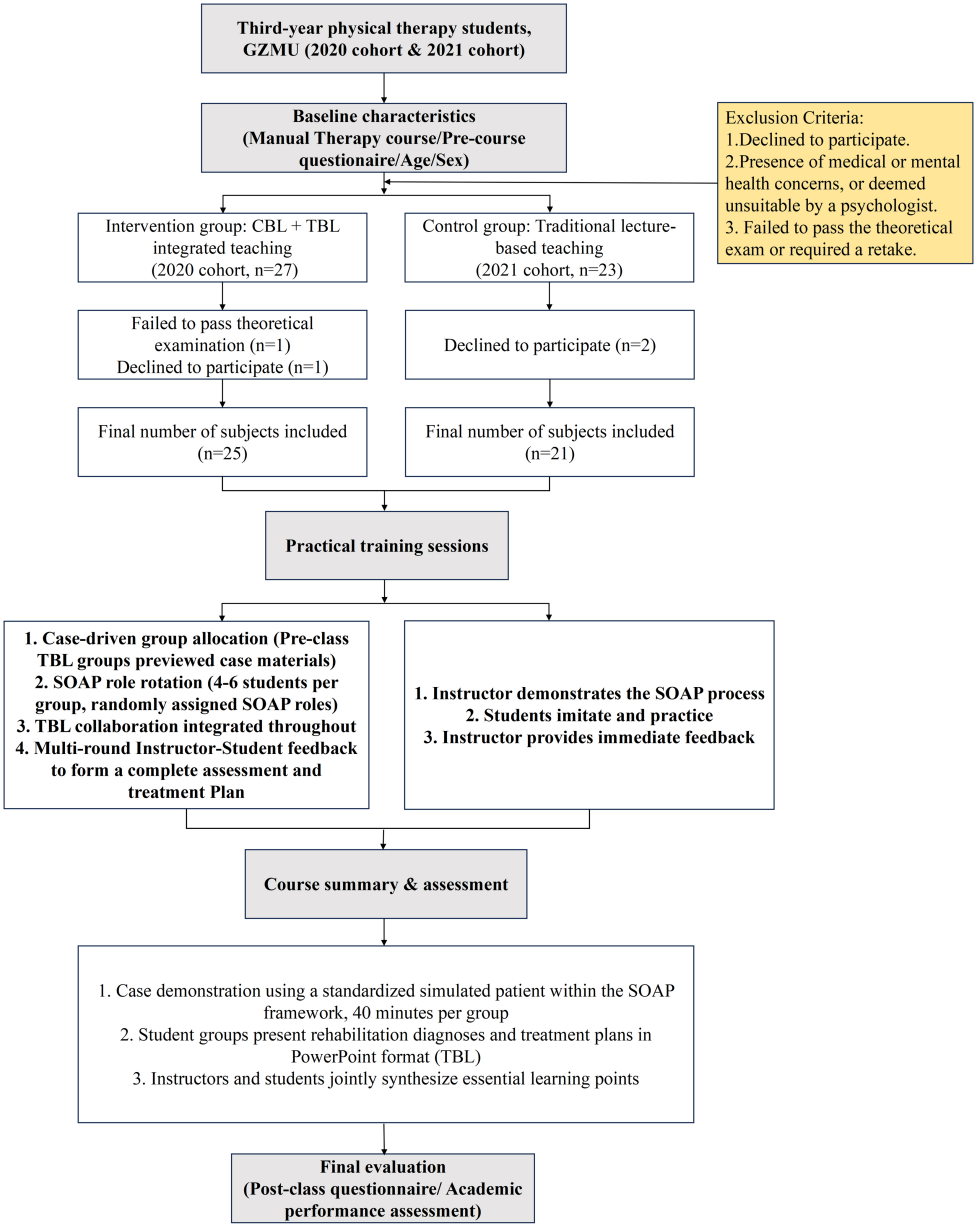
Study flowchart.

A quasi-experimental design was adopted in this study. The intervention group (2020 cohort) consisted of students who had received instruction using CBL integrated with TBL, whereas the control group (2021 cohort) consisted of students who had been taught through conventional lecture-based methods. Based on academic performance and questionnaire data routinely collected during the course instruction process, a comparative analysis of student learning outcomes under different teaching modes was performed from March 2023 to June 2024. The Ethics Committee of the Fifth Affiliated Hospital of Guangzhou Medical University (GZMU) approved the study (Ethics Approval Number: GYWY-L2024-81). The research adhered to the principles outlined in the Declaration of Helsinki. All included participants were re-contacted and provided written informed consent for the use of their educational data. The study flowchart is illustrated in Figure 1.

The inclusion criteria were: (1) third-year students from the 2020 and 2021 cohorts of the PT Department at GZMU’s Fifth Clinical College; (2) students who voluntarily participated and consented to complete the questionnaire. The exclusion criteria comprised: (1) students who opted out of participation; (2) students with documented medical or mental health concerns or those advised against participation by a psychologist; (3) students who failed to pass the theoretical examination or required a retake.

After excluding one student for failing the theoretical exam and one for refusal in the 2020 cohort, along with two refusals in the 2021 cohort, a total of 46 students (92% of the course enrollment) were included in the final analysis. To establish baseline comparability between the intervention and control groups, data collected prior to the commencement of the course were reviewed. Course grades in Manual Therapy, a subject directly related to PTOD, were retrieved, and pre-course evaluation scores using the questionnaire developed by Lee BF et al. (22) were extracted from academic records.

### 2.2 PTOD

This course focuses on PT for common orthopedic disorders of the lumbar spine, pelvic, hip, knee, ankle and foot. Centered on evidence-based practice, it systematically covers the latest clinical practice guidelines and scientific evidence for interventions targeting these regions. The course adopts the Subjective, Objective, Assessment and Plan (SOAP) clinical framework (23): the Subjective component emphasizes collecting detailed medical history and symptoms related to orthopedic dysfunction; the Objective component focuses on precise physical examinations and functional assessments, including active and passive joint range of motion, accessory movements, special tests, and neurosensory function; the Assessment phase integrates evidence-based analysis for clinical diagnosis; and the Plan phase develops individualized, goal-oriented PT interventions. With a strong emphasis on clinical practice, The course was designed to enhance students’ application of evidence-based knowledge and foster core competencies in clinical reasoning, problem-solving, and hands-on skills through intensive practical training. The course comprises 80 h, with 16 h of theoretical instruction and 64 h of practical training, ensuring comprehensive skill development and critical thinking (3).

### 2.3 Intervention

#### 2.3.1 2020 cohort (integrated CBL and TBL model)

The theoretical instruction was presented through PowerPoint lectures, integrating evidence-based rehabilitation principles, with educators facilitating students’ rigorous acquisition of rehabilitation knowledge relevant to specific scenarios. The instructional methodology was structured on CBL and TBL as its fundamental elements. All students were allocated to four permanent learning groups, each comprising 4–6 students, with group membership remaining unchanged throughout the course.

The practical teaching program was divided into seven modules, covering the thoracic spine, lumbar spine, pelvis, hip, knee, ankle, and foot, respectively. In the second session (4 contact hours) of each module’s practical teaching unit, SOAP-based instructional activities based on actual clinical cases were executed (CBL). The majority of case patients were drawn from the class. Students presenting with diseases or symptoms pertinent to the course were proactively recruited. The instructor distributed a recruitment announcement in class 1 week prior to identify appropriate situations. In the absence of suitable cases within the existing cohort, a fourth-year intern from the Fifth Affiliated Hospital of GZMU, possessing relevant disease characteristics, was recruited to maintain case authenticity and instructional continuity. All participants, including students from the class and the external intern, provided informed consent and subsequently assumed the role of genuine patients.

Five days before to each case-based teaching session, the four learning groups were randomly allocated specific responsibilities within the SOAP framework: Subjective (S), Objective (O), Assessment (A), and Plan (P). Focussing on the identical patient case information, each group worked beforehand to examine pertinent documents and perform analyses (TBL) from their designated SOAP perspective, in preparation for the in-class presentation. During the practical session, all groups conducted practical skills training and demonstrations with the actual patient case (integrated CBL + TBL). Roles were randomly redistributed for each case session to avert role fixation, guarantee equal participation, and promote a thorough comprehension of the entire clinical reasoning process.

As an illustrative example, a student-patient with low back pain related to lumbar spine pathology is described (CBL and TBL were implemented simultaneously during the SOAP sessions). The four fixed learning groups were assigned different SOAP components for analysis.

Group 1 was responsible for the Subjective (S) phase. Students meticulously conducted interviews with the patient to gather fundamental information and medical history, encompassing age, gender, hobbies, occupational attributes, familial context, personal income, capacity for consistent follow-up, precipitating factors such as prolonged sitting, heavy lifting, or lumbar rotation, duration of symptoms, characteristics and distribution of pain, exacerbating and alleviating factors, diurnal symptom fluctuations, previous treatment history, and possible contraindications. In this phase, Group 1 predominantly executed the interview, whereas Groups 2–4 documented faults, omissions, or unsuitable questioning techniques. Observational feedback was delivered at the faculty-student interaction subsequent to Group 1’s completion.

Group 2 was accountable for the Objective (O) phase. Students conducted examinations in accordance with the lumbar spine physiotherapy assessment protocol, encompassing imaging evaluation; static and dynamic visual inspection; soft tissue palpation; lumbar functional movement assessment; muscle strength and sensory evaluation; neurological reflex testing; accessory movements; and disease-specific special tests (e.g., straight leg raise test, Slump test), added as necessary by functional scales such as the Oswestry Disability Index (ODI). In this phase, Group 2 predominantly executed the objective examinations, while Groups 1, 3, and 4 documented procedural steps, operational specifics (e.g., palpation force, fixation sites, direction, and position), and the relevance of particular tests in relation to disease characteristics. Feedback was given during the ensuing faculty-student discussion.

Group 3 was tasked with the Assessment (A) phase. Students synthesized subjective and objective data to identify the primary etiology of low back pain and performed a physiotherapy assessment in accordance with the International Classification of Functioning, Disability and Health (ICF), distinguishing between potential disc-related complications, articular factors, myofascial issues, or postural and motor control deficits. They delineated principal issues from the standpoint of functional activity constraints and formulated both short-term and long-term objectives. Responses from other groups adhered to the aforementioned pattern.

Group 4 was accountable for the Plan (P) phase. Following the assessment results, the group formulated a preliminary physiotherapy plan encompassing physical agent modalities, joint mobilization, soft tissue manual techniques, core stability and motor control training, as well as health education with home exercise instructions aimed at daily activities and posture. Responses from other groups adhered to the aforementioned pattern.

#### 2.3.2 2021 cohort (traditional teaching model)

Theoretical instruction consisted of PowerPoint presentations delivered by the instructor. During practical sessions, the instructor first explained the core concepts and key issues (for approximately 30 min), demonstrated the SOAP process, and then had students practice each procedural step in small groups of 2–3, following the demonstrated model. The instructor provided feedback on operational details, and discussions were integrated throughout the session. In the final eight sessions of the course, students were required to deliver PowerPoint presentations, on which the instructor provided structured feedback. Both student cohorts were instructed by the same teaching team.

### 2.4 Evaluation

#### 2.4.1 Academic performance

Academic Academic performance was evaluated using a structured scoring system: class performance constituted 10%, practical performance 40%, and theoretical scores (written examination) 50%. If the theoretical score was under 60 points, it was directly utilized as the final overall grade. The content and difficulty of the theoretical examinations were similar throughout the two student cohorts.

The assessment criteria for academic and practical performance were the same for both groups. Class performance was predominantly evaluated based on students’ involvement in classroom activities, encompassing attendance, participation frequency in discussions and Q&A sessions, collaborative efficacy in group work (group scoring), and assignment grades. Instructors consistently assessed students’ classroom engagement during the instructional process and delivered a comprehensive evaluation by synthesizing their performance in group discussions and practical activities.

The practical performance involved a thorough standardized case demonstration utilizing the SOAP framework, executed at the conclusion of the course for both groups. Each group of 4–6 students (adopting the CBL + TBL grouping model for the 2020 cohort) was required to complete the designated case operation within 40 min. This evaluation concentrated on assessing students’ comprehension and application of the case, the logical reasoning underlying their clinical thought, and the standardization of their operating protocols.

#### 2.4.2 Questionnaire

This study employed a teaching effectiveness evaluation questionnaire as the research instrument to examine students’ subjective experiences and learning outcomes before and after the course (Appendix 1). The questionnaire comprised 64 standardized items, addressing six fundamental dimensions of the student learning process: Self-assessment (12 items), Self-directed learning (20 items), Group-cooperation (6 items), Teacher-assessment (3 items), Learning-effectiveness (10 items), and Learning-satisfaction (13 items). The questionnaire utilized a 5-point Likert scale (1 = “strongly disagree,” 5 = “strongly agree”), where elevated scores indicate enhanced observed impact in that dimension. The Cronbach’s *α* coefficients for the six dimensions of the revised scale were 0.79, 0.82, 0.88, 0.80, 0.91, and 0.95, confirming its overall reliability and validity (22).

### 2.5 Statistical analysis

Statistical analysis was performed using SPSS 27.0. Normality of variables was evaluated based on skewness (±2) and kurtosis (±7) thresholds recommended by Kline (24). Results showed that the pre-test and post-test total scores and all dimensional scores of the questionnaire, along with most academic performance measures (except pre-test class performance) and age, were normally distributed and were thus analyzed using independent or paired t-tests. Non-normal variables (pre-test class performance) were compared between groups with the Mann–Whitney U test, while gender distribution was assessed with the chi-square test. Data are presented as frequencies for categorical variables, mean ± SD for normally distributed continuous variables, and median (P25, P75) for non-normally distributed continuous variables. The significance level was set at *p <* 0.05.

## 3 Results

### 3.1 Baseline characteristics

A total of 46 students were ultimately incorporated into the study. The 2020 cohort had 6 male students (24%) and 19 female students (76%), with a mean age of 20.12 ± 0.526 years; the 2021 cohort contained 9 male students (42.9%) and 12 female students (57.1%), with a mean age of 20.10 ± 0.625 years. No statistically significant differences were detected between the two groups for gender, age, pre-questionnaire scores, or pre-course academic performance (all *p* > 0.05), suggesting equivalent baseline characteristics at the commencement of the course (Tables 1–3).

**Table 1:** Comparison of sex and age distribution between the two groups.

Variables	2020 cohort (*n* = 25)	2021 cohort (*n* = 21)	Test statistics	*p*
Male	6(24.0)	9(42.9)	*χ*^2^ = 1.847	0.174
Female	19(76.0)	12(57.1)		
Age, year	20.12 ± 0.526	20.10 ± 0.625	*t* = 0.146	0.885

Categorical variables are showed as *n* (%); continuous variables with normal distribution are reported as mean ± SD.

**Table 2:** Comparison of baseline (pre-test) questionnaire scores between the two groups.

Variables	Total scores		Self-assessment		Self-directed learning		Group-cooperation		Teacher-assessment		Learning-effectiveness		Learning-satisfaction	
Group	2020 cohort	2021 cohort	2020 cohort	2021 cohort	2020 cohort	2021 cohort	2020 cohort	2021 cohort	2020 cohort	2021 cohort	2020 cohort	2021 cohort	2020 cohort	2021 cohort
*N*	25	21	25	21	25	21	25	21	25	21	25	21	25	21
M ± SD	198.32 ± 25.49	196 ± 18.55	34.76 ± 8.90	34.29 ± 8.25	57.04 ± 14.32	56.76 ± 10.00	19.12 ± 4.99	19.05 ± 4.60	9.08 ± 2.99	9.38 ± 1.47	36.16 ± 6.32	35.48 ± 5.11	42.16 ± 9.53	41.05 ± 7.36
Test statistics	t= 0.347		t= 0.186		t= 0.075		t= 0.051		t= −0.444		t= 0.399		t= 0.437	
*p*	0.730		0.853		0.941		0.960		0.659		0.692		0.665	

M, mean; SD, standard deviation.

**Table 3:** Comparison of basic academic performance between the two groups.

Variables	Class performance		Practical performance		Theoretical scores		Overall grades	
Group	2020 cohort	2021 cohort	2020 cohort	2021 cohort	2020 cohort	2021 cohort	2020 cohort	2021 cohort
N	25	21	25	21	25	21	25	21
M ± SD /Median(P_25_, P_75_)	95(93.5,96)	93(92,95)	89.97 ± 1.96	89.95 ± 1.99	80.24 ± 5.40	77.67 ± 5.06	85.84 ± 3.10	84.38 ± 2.81
Test statistics	Z = -2.517^b^		t = 0.034^a^		t = 1.656^a^		t = 1.655^a^	
*p*	0.012		0.973		0.105		0.105	

M, mean; SD, standard deviation; ^a^Independent samples t-test, ^b^Mann-Whitney U test.

### 3.2 Between-groups analysis of post-course outcomes

**Figure 2:**
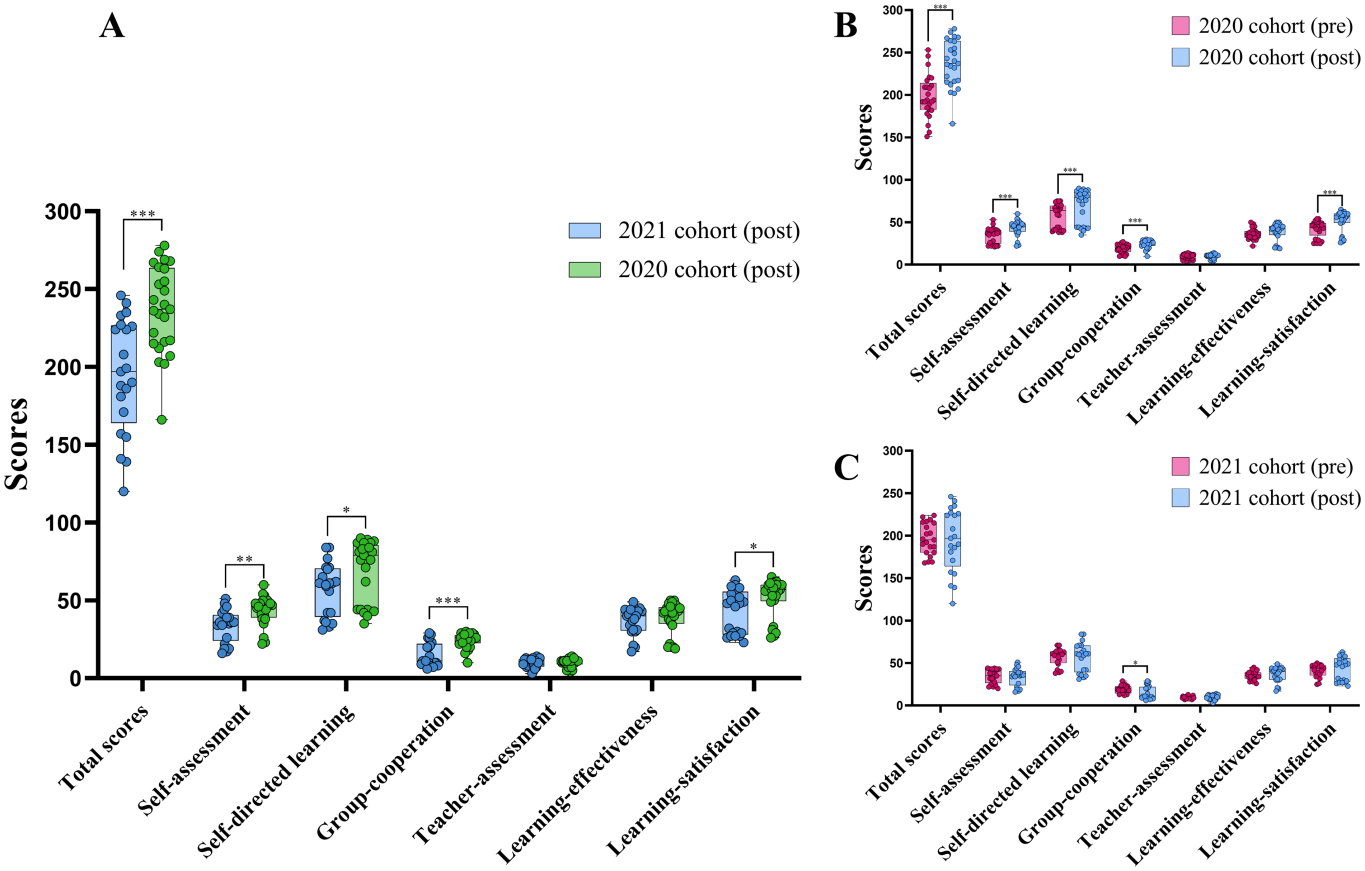
Questionnaires between groups and within group. **(A)** Between-groups analysis of post-course outcomes. **(B)** Within-groups analysis of 2020 cohort outcomes. **(C)** Within-groups analysis of 2021 cohort (***p < 0.001, **p < 0.01, *p < 0.05).

The results (Figure 2A) revealed significant differences in total scores of the questionnaires between groups (*p* < 0.001), with the 2020 cohort scoring significantly higher than the 2021 cohort. In sub-dimension comparisons, the intervention group outperformed the control group in Self-assessment (*p* < 0.01), Self-directed learning (*p* < 0.05), Group-cooperation (*p* < 0.001), and learning satisfaction (*p* < 0.05).

### 3.3 Within-groups analysis of 2020 cohort outcomes

The pre-post mean difference (MD = Post - Pre) in total scores for the 2020 cohort was significant (*p* **<** 0.001), with post-test scores significantly higher than pre-test scores. Across sub-dimensions, significant improvements were observed in Self-assessment (*p* < 0.001), Self-directed learning (*p* < 0.001), Group-cooperation (*p* < 0.001), and Learning-satisfaction (*p* < 0.001). However, no significant differences were found in Teacher-assessment or Learning-effectiveness dimensions (*p* > 0.05; Figure 2B).

### 3.4 Within-groups analysis of 2021 cohort outcomes

The pre-post comparison of questionnaire findings for the control group (2021 cohort) indicated a slight decline in total scores and Self-assessment scores at post-test relative to pre-test; however, these differences were not statistically significant (*p* > 0.05). Group-cooperation scores among sub-dimensions showed a substantial reduction (*p* < 0.05), indicating statistical significance. Post-test scores for Self-directed learning, Teacher-assessment, Learning-effectiveness, and Learning-satisfaction exceeded pre-test values; nevertheless, none exhibited statistically significant differences (*p* > 0.05; Figure 2C).

### 3.5 Post-course academic performance between groups

The results of the performance comparison (Table 4) indicated that, no statistically significant difference in overall grades between the two cohorts (*t* = −0.28, *p* = 0.781). However, the 2020 cohort significantly outperformed the 2021 cohort in class performance (*t* = 3.253, *p* = 0.002) and practical performance (*t* = 2.581, *p* = 0.013). No statistically significant difference was observed in the final theoretical scores between the two groups (*p* > 0.05).

**Table 4:** Comparison of post-test academic performance between the two groups.

Variables	Class performance		Practical Performance	Theoretical scores			Overall grades	
Group	2020 cohort	2021 cohort	2020 cohort	2021 cohort	2020 cohort	2021 cohort	2020 cohort	2021 cohort
N	25	21	25	21	25	21	25	21
M ± SD	94.68 ± 2.56	92.43 ± 2.038	92.68 ± 2.19	90.76 ± 2.84	74.24 ± 5.16	76.57 ± 4.44	83.76 ± 2.89	84.00 ± 2.90
Test statistics	t= 3.253		t= 2.581	t= −1.625			t= −0.28	
*p*	0.002		0.013	0.111			0.781	

M, mean; SD, standard deviation.

## 4 Discussion

Our findings suggest that the integrated CBL + TBL model may serve as a promising pedagogical approach to address the pervasive challenges of student passivity and underdeveloped clinical reasoning in traditional PT education. By creating a structured, interactive, and student-centered learning environment, the integrated CBL + TBL model was associated with significantly higher levels of students’ Self-directed learning, Group-cooperation, and Learning satisfaction, key drivers of professional development in healthcare. These results indicate that this pedagogical approach may help address several issues inherent in traditional teaching methods, including insufficient student engagement, lack of motivation for self-directed learning (5), and inadequate development of clinical reasoning skills (6). Specifically, students in the CBL + TBL group demonstrated significantly higher scores in learning motivation and clinical reasoning abilities, potentially attributable to increased classroom interaction frequency and self-directed learning time. These findings provide preliminary support for the practical feasibility of the “student-centered” teaching philosophy in PT education.

### 4.1 The efficacy of the CBL + TBL model

The results of this study are partially consistent with existing literature, which suggests that TBL effectively enhances learning responsibility and classroom participation (10), while CBL strengthens students’ clinical reasoning and diagnostic capabilities (19, 20). Building on this, the study explored the application of the CBL + TBL blended teaching model in the PTOD course, with findings indicating favorable outcomes in the intervention group. The observed benefits of the CBL + TBL model may stem from a synergistic cycle, rather than a mere summation of two methods. First, TBL’s pre-class preparation encourages students to acquire foundational knowledge, potentially providing a ‘theoretical scaffold’ for subsequent case analysis (25). Subsequently, CBL may provide a meaningful context for applying this knowledge to complex, authentic clinical cases, thereby bridging the theory-practice gap (20, 26). Furthermore, the team discussions in TBL may foster the collaborative skills essential for effective case deliberation in CBL (5). Finally, the role-specific group tasks within CBL cases demand individual accountability, which in turn may reinforce the positive interdependence cultivated in TBL. This potentially creates a self-reinforcing loop of knowledge acquisition, application, collaboration, and accountability.

Additionally, this study integrated the SOAP structured framework (27) as a vehicle for implementing the CBL + TBL model, aiming to bridge theoretical learning and practical training. In CBL + TBL classrooms, students collaborated in groups around real clinical cases, performing role-specific tasks within the SOAP framework, which was designed to ensure deep engagement in distinct aspects of the clinical reasoning process. This structured, phased, task-driven teaching approach may have facilitated learning progression (28), promoted student initiative, and supported the development of comprehensive analytical and judgment skills (29, 30). Notably, the SOAP framework served as a core scaffold, potentially integrating CBL and TBL into a cohesive clinical reasoning process, extending beyond a mere documentation tool.

### 4.2 Academic performance

A particularly noteworthy finding is the dissociation between practical and theoretical performance. The intervention group demonstrated significantly superior practical skills and class performance despite comparable theoretical exam scores. This differential effect provides support for the model’s premise: it may more effectively enhance the ‘know-how’ (practical competence and clinical application) without compromising the ‘know-what’ (declarative knowledge). For a highly pragmatic field like PT, where competency is ultimately defined by performance (3), this shift toward enhancing practical prowess is arguably more meaningful than gains on standardized written tests.

Moreover, no significant differences were observed in teacher evaluations or overall academic grades between the two groups. The absence of a significant difference in overall academic performance warrants careful interpretation. While it might suggest comparable efficacy in knowledge transfer, it more likely indicates that the standardized written examinations used in this study may be insensitive to the higher-order cognitive skills potentially fostered by the CBL + TBL model, such as clinical reasoning and adaptive problem-solving. On the one hand, the comparable scores suggest that the integrated teaching model was not associated with any decline in student performance, supporting its potential to maintain standardized assessment outcomes (31). On the other hand, the similar scores on the Teacher-assessment dimension, which comprised only three items, may not reflect a true equivalence of teaching quality but rather the limitations of the evaluation tool itself. Generic satisfaction surveys often fail to discern nuances between pedagogical approaches. This finding suggests that teachers and medical education institutions should comprehensively consider disciplinary characteristics and students’ demographic factors when designing hybrid teaching models (18). Further efforts are needed to strengthen the integration and summarization of foundational knowledge and to ensure effective alignment between theoretical frameworks and clinical reasoning to optimize teaching outcomes without compromising the overall student learning experience.

### 4.3 Learning satisfaction

Notably, learning satisfaction, as a subjective indicator, was significantly higher in the CBL + TBL group, suggesting that this model positively impacted students’ classroom experience. This result can be attributed to the interactive, task-oriented design of the model, which fosters a friendly competitive environment within groups, facilitating timely instructor feedback during class (32), and enhancing students’ sense of responsibility toward their peers (33). Such an environment encourages greater engagement in pre-class preparation and classroom activities. In highly practical courses such as PTOD, this model appears particularly effective in stimulating active student participation (16). Moreover, the SOAP role-rotation mechanism required students to simulate various clinical roles through phased tasks and collaboration. This approach not only strengthened the integration and application of practical skills but also deepened students’ understanding of structured clinical reasoning logic (34). These findings align with those of Burgess et al. (28), who emphasized that the integration of CBL and TBL promotes the transfer of foundational knowledge and the development of clinical reasoning skills, thereby enhancing learning efficiency and intrinsic motivation. Thus, this teaching design likely improves students’ learning satisfaction and sustained engagement by addressing their needs for autonomy, competence, and relatedness.

### 4.4 Recommendations

Based on our findings, we propose the following recommendations for implementing the CBL + TBL model: First, the integrated teaching design may be optimized. The case difficulty and TBL tasks should align with students’ cognitive foundation (19, 28), and the introduction of highly simulated clinical scenarios may help systematically enhance students’ clinical reasoning and problem-solving abilities in ways that approximate practice (16). Second, task design may follow the principle of gradual progression, ensuring that students master core knowledge (17) while maintaining an appropriate cognitive load (10). Future studies could incorporate assessment tools specifically designed to evaluate clinical reasoning to more comprehensively capture the model’s potential impact. Third, teachers may need to anticipate and mitigate possible cognitive overload associated with the mixed mode, while providing guidance in team collaboration and metacognitive strategies (5, 32).

### 4.5 Limitations

The interpretation of our findings should be considered in light of several limitations. First, the quasi-experimental design conducted at a single institution, while logistically necessary, limits the generalizability of the findings. Future multicenter, large-sample studies would be valuable to further examine the external validity of this model. Second, the assessment of teaching effectiveness relied primarily on student self-reported scales, without objective measures of practical competency (e.g., OSCE). Future studies could incorporate diverse objective evaluation metrics to strengthen the assessment system. Additionally, this study assessed only the short-term outcomes of the teaching intervention; therefore, longitudinal research would be an important direction for future investigation.

As a quasi-experimental study, a fundamental limitation is the lack of random assignment of participants. Despite the implementation of several procedures to improve comparability between groups, numerous potential confounding factors require consideration. Both groups received instruction from the same teaching team using similar content and curriculum to mitigate instructor variability. All sessions were conducted physically, assuring the stability of the instructional environment. Regarding baseline student characteristics, while academic performance was statistically controlled, unmeasured variables such as prior clinical experience and intrinsic motivation may still vary between groups. Despite the utilization of standardized criteria and evaluators in the assessment process, inter-rater subjective bias cannot be completely eradicated. The design decisions were to reduce confounding variables and improve internal validity. Subsequent research may implement a randomized controlled design and include pre-test matching based on prior knowledge, motivation, and other characteristics to yield more substantial evidence.

## 5 Conclusion

This study suggests that the CBL + TBL hybrid model, through the synergistic integration of case-driven learning and team collaboration within the SOAP framework, may help bridge the gap between theory and clinical practice in the PTOD course. In addition to being associated with improvements in students’ self-directed learning motivation, clinical reasoning abilities, and teamwork competencies, the model also appeared to show a dissociation between practical skill improvement and theoretical test performance, highlighting its potential value in cultivating core clinical competencies.

## Data Availability

The original contributions presented in the study are included in the article/Supplementary material, further inquiries can be directed to the corresponding author/s.
