# Supplementary material for: Efficacy of an integrated case-based learning and team-based learning model within the subjective, objective, assessment, and plan framework in physical therapy education
Source: Front Med (Lausanne). 2026 Feb 19;13:1767677. doi: 10.3389/fmed.2026.1767677 (PMC12960088; doi:10.3389/fmed.2026.1767677)
Supplement: Supplementary file 1 [file Table_1.doc]

**Appendix 1**

The 64-item scale.

| **self-assessment (12 items)** | |
| --- | --- |
| 1 | Please rate yourself from being quiet to being lively. |
| 2 | Please rate yourself from being absently minded to being minded. |
| 3 | Please rate yourself from being calm to being restless. |
| 4 | Please rate yourself from listening to exploring. |
| 5 | Please rate yourself from obeying to leading somebody. |
| 6 | Please rate yourself from thinking to operating. |
| 7 | Please rate yourself from describing something by words to by image. |
| 8 | Please rate yourself from reasoning to acting on instinct. |
| 9 | Please rate yourself from solving problems yourself to discussing with others. |
| 10 | Please rate yourself from solving problems passively to doing actively. |
| 11 | Please rate yourself from solving problems in a daze to exploring them. |
| 12 | Please rate yourself from solving problems by systematic processing to being tentative. |

| **self-directed learning (20 items)** | |
| --- | --- |
| 1 | I like group learning. |
| 2 | I like to share knowledge and experience with the classmates. |
| 3 | I listen to other people's ideas and suggestions. |
| 4 | I accept the comments of somebody. |
| 5 | I am an emotional person. |
| 6 | I am able to think rationally. |
| 7 | I can talk to strangers. |
| 8 | I cannot express myself well. |
| 9 | I trust my own judgment. |
| 10 | I understand my personality. |
| 11 | I am good at analyzing and reasoning. |
| 12 | I am good at integrated coordination. |
| 13 | To stimulate more ideas with the students worked in a group. |
| 14 | I am confident with regard to make a presentation. |
| 15 | I often have no idea how to talk in group discussion. |
| 16 | I am always getting *distracted* in group discussion. |
| 17 | I often cannot get the currently focused element in group discussion. |
| 18 | I usually do not know how to ask questions in response to the classmate’s oral presentation. |
| 19 | I am usually skeptical about the classmate’s report. |
| 20 | I often cannot find the right information to make the oral presentation. |

| **group-cooperation (6 items)** | |
| --- | --- |
| 1 | The frequency of the group discussion is appropriate. |
| 2 | The frequency of everyone to make an oral statement is appropriate in group discussion. |
| 3 | The number of members in one group is appropriate. |
| 4 | A small number of classmates have the much time to talk in group discussion. |
| 5 | The contents are not focused in group discussion. |
| 6 | It is efficient for the analysis of the problems in group discussion. |

| **teacher-assessment (3 items)** | |
| --- | --- |
| 1 | The teacher in the class is serious in group discussion. |
| 2 | The teacher often interrupts the discussing process in group discussion. |
| 3 | The teacher has good interaction with students in group discussion. |

| **learning-effectiveness (10 items)** | |
| --- | --- |
| 1 | CBL is better than TIA. |
| 2 | CBL process enabled you to access a greater variety of resources. |
| 3 | CBL is an efficient learning method. |
| 4 | You can achieve the intended learning objectives in each group discussion. |
| 5 | CBL process enabled you to easily understand the problems of every case. |
| 6 | The comments of classmates had great reference value on analyzing the problems. |
| 7 | CBL process improved your ability to solve professional problems. |
| 8 | CBL process is efficient for integrating the important information. |
| 9 | CBL process improved your interpersonal skills to solve problems by teamwork. |
| 10 | CBL process has improved your clinical reasoning skills and clinical competence independently. |

| **learning-satisfaction (13 items)** | |
| --- | --- |
| 1 | CBL process is a challenge for me in this semester. |
| 2 | CBL method improved your ability of gathering information , organizing them |
|  | and store in usable form for future use |
| 3 | CBL method has motivated me to learn |
| 4 | After CBL method, I am confident for my analysis work. |
| 5 | After CBL method, I have better skills to solve the problems. |
| 6 | Overall during our discussion, faculty I approached was very helpful and the discussion has helped me in learning. |
| 7 | After CBL method, I can find out the reasons to support or oppose the problems. |
| 8 | Overall during our discussion, I learned how to lead the team to do the discussion. |
| 9 | Overall during our discussion, I learned how to do self-integration and construct knowledge by myself. |
| 10 | I have better interpersonal skills in terms of listening, giving, and receiving criticism etc. |
| 11 | After CBL method, I learned how to do more in-depth data analysis. |
| 12 | I find that the CBL process has changed the way I study. |
| 13 | Please rate the CBL method. |
